# Detection of *Mycobacterium tuberculosis* and rifampicin resistance by Xpert^®^ MTB/RIF assay among presumptive tuberculosis cases at Jimma University Medical Center, Southwest Ethiopia

**DOI:** 10.1371/journal.pone.0262929

**Published:** 2022-01-27

**Authors:** Wasihun Admassu, Birhanu Ayelign, Gemeda Abebe, Mulualem Tadesse

**Affiliations:** 1 Immunology and Molecular Biology Unit, Jimma University Medical Center, Jimma, Ethiopia; 2 Department of Immunology and Molecular Biology, School of Biomedical and Laboratory Science, College of Medicine and Health Science, University of Gondar, Gondar, Ethiopia; 3 Mycobacteriology Research Center, Jimma University, Jimma, Ethiopia; 4 School of Medical Laboratory Sciences, Faculty of Health Sciences, Jimma University, Jimma, Ethiopia; Indian Institute of Technology Delhi, INDIA

## Abstract

**Background:**

Rapid diagnosis of tuberculosis (TB) and detection of drug resistance are very important for timely and appropriate management of patients. Xpert MTB/RIF assay is approved for use in TB and rifampicin-resistance diagnosis. However, data are limited on the impact of Xpert MTB/RIF assay under routine clinical settings with a heterogeneous group of patients and sample types in Ethiopia.

**Methods:**

A retrospective study was carried out in 2220 presumptive TB cases at Jimma University Medical Center. Data were gathered from the registration logbook using formatted data extraction tools and double entered to epidata version 3.1 and further transported to SPSS version 20 for analysis. Associations were determined using the Chi-square test and *P-value* <0.05 was considered statistically significant.

**Results:**

Of 2220 cases enrolled, 1665 (75%) were adults and the remaining 555 (25%) were children aged less than 14 years. The majority, 1964 (88.46%), had pulmonary manifestation and 256 (11.54%) had extrapulmonary involvements. The overall, frequency of *Mycobacterium tuberculosis* (MTB) was 9.3% (206/2220), among this 10.27% (171/1665) and 6.3% (35/555) were adults and children, respectively. *M*. *tuberculosis* was detected from 171 (8.75%) of pulmonary patients and 35 (13.28%) of extrapulmonary manifested patients. Out of 206 *M*. *tuberculosis* positive cases, 7(3.4%) were rifampicin-resistant: four from pulmonary tuberculosis (PTB) patients and three from EPTB patients. In the Chi-square test, the age group of 15–24 years, previous history of TB, pus/lymph node sample, and being HIV positive were significantly associated with TB positivity by Xpert MTB/RIF (*P-value* <0.001).

**Conclusion:**

These data suggest that the overall frequency of *M*. *tuberculosis* and rifampicin resistance was found to be relatively low compared to the previous reports in Ethiopia. Nevertheless, better diagnostic tools and approaches are still vital to halt the burden of TB and drug-resistant TB in the country.

## Background

Tuberculosis (TB) remains the leading cause of morbidity and mortality throughout the world. According to the 2019 WHO report, over 10 million people are estimated to have TB morbidity with 1.3 million deaths. Among those, 90% belong to reproductive age groups and 9% of TB patients had TB/HIV co-infection [1]. Nowadays, an increased multidrug resistance TB (MDR-TB) cause a big challenge to global and national TB control program. About 82% of MDR-TB were detected from 558,000 cases of rifampicin resistant TB (RR-TB) cases in 2018 [1]. Moreover, empirical TB treatment is suggested to increase the risk of transmission of drug-resistant strains [2]. Ethiopia is one of high TB, TB/HIV, and MDR-TB burden countries possessing an incidence rate of 164, 12, and 5.5 per 100,000 population, respectively [1].

One of the pillars in the implementation of stop TB strategy is the early diagnosis of TB with their drug sensitivity test. However, this early detection has been hindered by poor sensitivity of conventional diagnostic tools particularly on smear-negative individuals, specimen other than sputum, and immune-compromised persons. In 2010, WHO endorsed the Xpert MTB/RIF assay, which is a rapid and automated molecular system that detects both *M*. *tuberculosis* (MTB) and its resistance to rifampicin. The Xpert MTB/RIF assay is a cartridge-based, automated diagnostic test that can identify MTB and rifampicin-resistance in less than 2 hours. It requires simple technical expertise and can detect TB and rifampicin resistance in pulmonary and extrapulmonary clinical samples [3–6]. In recent experiments, Xpert MTB/RIF assay showed superiority in the identification of cases compared to conventional methods and culture in smear negatives cases [3, 5]. Hence, after the endorsement of Xpert MTB/RIF assay by WHO in 2010, an immense improvement in case detection was observed [1, 7, 8].

Previous studies in our study setting showed high prevalence of smear-negative, pediatric and extrapulmonary TB cases [6, 9, 10]. However, these previous studies have not dealt with respect to sample types, large sample size, drug sensitivity tests, and comparison to a pediatric and adult subpopulation. In addition, no research has been found that surveyed the magnitude of TB in our setting by Xpert MTB/RIF assay with respect to rifampicin resistance patterns in the heterogeneous study populations [3, 11, 12]. Thus, the present study provides additional evidence with respect to the overall prevalence of TB, pulmonary TB, and EPTB with its drug susceptibility pattern in different sample types in Jimma Medical Center which services as the major referral center for Southwest Ethiopia.

## Methods and materials

### Study design and setting

An institution-based retrospective study was conducted from May 2018 to August 2019 at Jimma Medical Center (JMC) which is located in Southwest Ethiopia. It is one of the oldest public hospitals in the country which was established in 1930 E.C. Jimma Medical Center is a specialized and referral hospital that provides services for about 15 million populations in the catchment area. It offers service for approximately 15, 000 inpatients, 160, 000 outpatient attendants, and 11, 000 emergency cases annually. Jimma Medical Center Laboratory is one of the biggest service laboratory centers in-country and composed of the following units; Molecular and Immunology, Clinical Chemistry, Hematology, Immunohematology, Microbiology, Mycology and Virology, and Parasitology service. The center laboratory has a separate Xpert MTB/RIF assay section composed of two Xpert MTB/RIF assay machines which have 8 sample analysis potential at a time.

### Diagnosis of TB and rifampicin resistance at JMC

WHO has recommended, in 2011, starting roll-out of the Xpert MTB/RIF assay system in countries to improve TB diagnosis and detection of rifampicin resistance. Following the WHO recommendation, the Ethiopian National TB Control Program has developed an implementation guideline on the use of Xpert MTB/RIF assay in Ethiopia. Thus, in our hospital, Xpert MTB/RIF assay has been used as a primary diagnostic tool to diagnose TB and rifampicin resistance among presumptive TB cases. Clinical samples (pulmonary and extrapulmonary) were collected from individuals suspected of having TB and rifampicin resistance. Xpert MTB/RIF assay was performed on all sample types following the manufacturer instruction [13, 14]. Briefly, the clinical samples were diluted with sample reagent, vortexed, and kept for 15 minutes. Then, 2ml of the mix was transferred to Xpert cartridge and loaded onto the Xpert machine (Cepheid, Dx System Version 4.0c). Results were reported as detected, not detected or invalid/error for MTB. Likewise, rifampicin resistance results were reported as susceptible, resistant, or indeterminate.

### HIV testing

HIV testing was done according to the national algorithm recommended by the Federal Ministry of Health of Ethiopia. HIV (1+2) Antibody test (HIV 1/2 STAT-PAK (Chembio Diagnostics, USA) kit is run first on whole blood, plasma, or serum. Non-reactive result routinely announced as negative while positive outcomes tested for the second test (ABON HIV 1/2/O Tri-Line Human Immunodeficiency Virus Rapid Test Device (Abon biopharma (Hangzhou) co. Ltd, China)). Nonetheless, if both the first and the subsequent test becomes positive, they will be affirmed by third-line test (The SD BIOLINE HIV-1/2 3.0 test (SD standard diagnostics, INC, Republic of Korea)) and revealed as positive while in the event that it cannot help contradicting the past tests the outcomes will be considered as uncertain. Lastly, those with this inconclusive result will be retested after a period of time (three months).

### Data collection procedures

Data were collected retrospectively from registration books at Xpert MTB/RIF assay section of Jimma Medical Center using a data-extraction format after checking the completeness of the data. Data such as patient’s sex, age, sample types, category, and HIV status Xpert MTB/RIF assay results were extracted for each presumptive TB patient diagnosed using Xpert MTB/RIF assay during the course of the study year (May 2018 to August 2019). And patients with incomplete laboratory findings were excluded. The data were collected by trained laboratory personnel working at Xpert MTB/RIF assay section.

### Data analysis

The data were double entered and cleaned using Epi Data 3.1 (Jens M. Lauritsen & Michael Bruus) and transferred to SPSS version 20 (IBM, New York, and U.S). Results with ‘‘indeterminate” Xpert results (‘‘error”, ‘‘invalid” or ‘‘no result”) were excluded from the data extraction document. Data were analyzed using SPSS version 20. The results were summarized using descriptive statistics: percentiles, proportions, frequency distribution by tabulation. Bivariate statistical analysis was conducted to assess the association between each independent variable with their dependent variable using a chi-square test. A *P-value* < 0.05 was considered statistically significant.

### Ethics approval and consent to participate

Ethical clearance was obtained from institutional review boards of Jimma University, Ethiopia.

A letter of permission to conduct the study was obtained from Jimma Medical Center Laboratory. Informed consent was not sought from the study participants as it used secondary data from registration logbook. Confidentiality of the information collected was maintained information concerning the patients was kept confidential.

## Results

### Socio-demographic and clinical characteristics

A total of 2220 presumptive TB cases diagnosed from May 2018 to August 2019 by Xpert MTB/RIF assay were included in the study. The majority, 54.8% (1217), of participants were males. Female to male ratio of study participants was 1:1.2. Half of the participants were urban dwellers. Our study participants’ samples consist of 75.6% (1679) sputum and 12.5% (285) gastric lavage and 11.5% (256) extra pulmonary samples. The later includes pleural fluid (n = 88), CSF (n = 67), ascetic fluid (n = 7), puss (n = 35), lymph node (n = 8), genitourinary (n = 1), and peritoneal fluids (n = 50). A quarter (555) of our study groups were aged below 14 years and, 1.6% (36) had the previous history of TB treatment. Of the 1121 study participants who were tested for HIV, 8.6% (96) were HIV-positive. Out of the 2220 clinical samples analyzed by Xpert MTB/RIF assay, 9.3% (206) were MTB positive. Resistant to rifampicin was detected in 3.4% (7) of MTB positive cases (Table 1).

**Table 1:** Socio-demographic and clinical characteristic of study participants, from May 2018 to August 2019 at Jimma Medical Center, Southwest Ethiopia.

Variables	Classification	N = 2220	Percentage (%)
Sex	Male	1217	54.8
	Female	996	44.9
	Unspecified	7	0.3
Age groups	0 to 14yrs	555	25
	15 to 24yrs	324	14.6
	25 to 34yrs	383	17.3
	35 to 44yrs	350	15.8
	45 to 54yrs	276	12.4
	55 to 64yrs	168	7.6
	≥ 65yrs	164	7.4
Residence	Urban	1107	49.9
	Rural	758	34.1
	Unspecified	355	16
Previous TB history	Yes	36	1.6
	No	2150	96.8
	Unspecified	34	1.5
HIV status	Negative	1025	46.2
	Positive	96	4.3
	Unknown	1099	49.5
Sample type	Sputum	1679	75.6
	Gastric aspirates	285	12.8
	pleural fluid	88	4
	cerebrospinal fluid	67	3
	Ascetic fluid	7	0.3
	Puss (abscess)	35	1.6
	lymph node	8	0.4
	Genitourinary	1	0.00045
	Peritoneal	50	2.3
TB status	MTB Negative	2014	90.72
	MTB positive	206	9.28
TB positivity (PTB vs EPTB)	PTB detected, RIF sensitive	167	7.6
	PTB detected, RIF resistance	4	0.2
	EPTB detected, RIF sensitive	32	1.4
	EPTB detected, RIF resistance	3	0.1

### Magnitude of overall MTB, PTB, and EPTB

The overall prevalence of TB in the study was 9.3% (206). Of the 206 TB positive patients, 83% (171) were adults and the remaining 17% (35) were children. The TB positivity rate was 6.3% (35) among TB presumptive children and 10.3% (171) among the adult presumptive cases. In terms of the forms of TB, 8.7% (171) pulmonary TB presumptive cases and 13.7% (35) EPTB presumptive cases were confirmed to have MTB by Xpert MTB/RIF (Table 1).

### MTB positivity by sex, age group and residence

Of the study participants, 54.8% (1217) were males. However, the gender difference was not statistically significant (*p*. *value* > 0.436) with TB positivity rate. Out of 206 TB patients, 27.7% (57/206) were populated in the age range of 15–24 followed by age ranges of 25–34 and 35–44 each possessing 39 and 36 cases respectively. Of TB positive cases, 6.3% (35) were from children (<14years) and 10.3% (171) were from adolescents and adults (>14 years old). In this study, there was a significant association (*P*. *value* <0.0001) between MTB positivity and age groups, while gender and residence type didn`t associate with MTB positivity (Table 2).

**Table 2:** Mycobacterium tuberculosis (MTB) positivity among study participants based on associated variables (age, previous TB history, sample type and HIV status).

Variables		Xpert MTB/RIF assay result		Total (%)	P.VALUE
		positive	negative		
**Age**					< 0.001
	0–14	35(17.1)	520(25.8)	555(25)	
	15–24	57(27.3)	267(13.3)	324(14.6)	
	25–34	39(19)	344(17.1)	383(17.3)	
	35–44	36(17.6)	314(15.6)	350(15.8)	
	45–54	16(7.8)	260(12.9)	276(12.4)	
	55–64	13(6.3)	155(7.7)	168(7.6)	
	> = 65	10(4.9)	154(7.6)	164(7.4)	
	Total(% of total)	206(9.3)	2015(90.8)	2220(100)	
**History of TB treatment**					<0.001
	Previously treated	15(7.3%)	21(1.0%)	36(1.6%)	
	new	187(90.7%)	1963(97.5%)	2150(96.8)	
	Unknown	4 (2.0%)	30 (1.5%)	34 (1.5%)	
	total	206 (9.2)	2015(98.8)	2220(100)	
**sample types**					<0.001
	sputum	149(72.2)	1530(76)	1679(75.6)	
	Gastric aspirate	20(9.8)	265(13.2)	285(12.8)	
	pleural fluid	3(2)	85(4.2)	88(4)	
	CSF	4(2.4)	63(3.1)	67(3)	
	puss	20(9.8)	15(0.7)	35(1.6)	
	peritoneal fluid	2(2.9)	48(2.4)	50(2.3)	
	lymph node	6(2.9)	2(0.1)	8(0.4)	
	genitourinary	0	1(0.000)	0	
	Ascitic fluid	0	7(0.3)	7(0.3)	
	total	206(9.3)	2014(90.7)	2220(100)	
**HIV status**					<0.001
	Negative	74(35.9)	951(47.2)	1025(46.2)	
	Positive	33(16)	63(3.1)	96(4.3)	
	Unspecified	0	9(0.4)	9(0.4)	
	Unknown	99(48.1)	991(49.2)	1090(49.1)	
	Total	206(9.3)	2014(90.7)	2220(100)	

### MTB positivity by HIV status and previous TB history

HIV test result was available for 50.5% (1121) of the study participants. Of whom, 8.6% (96) were HIV positive. The remaining 45.5% (1099) of participants had no HIV test results documented. Of 96 HIV positive individuals, 34.4% (33) of them were TB positive by Xpert MTB/RIF assay. Likewise, of 36 patients with a previous history of TB treatment, 41.7% (15/36) were TB-positive compared to patients without a previous history of TB treatment, 8.7% (187/2150). In this study, there was a statistically significant association between the two conditions (being HIV-positive and having a previous history of TB) with MTB positivity rate (*P*. *value* < 0.001) (Table 2).

### MTB positivity by sample types

A total of 1964 pulmonary samples (1679 sputum and 285 gastric lavages) and 256 extrapulmonary samples were analyzed in this study. Sputum was the most commonly (75.6%) analyzed samples type followed by gastric lavage (12.8%), pleural fluids (4%), CSF (3%) and other sample types (peritoneal, pus, lymph node, ascetic fluid and urine) collectively accounting for 4.6% (Table 3). However, Xpert MTB/RIF MTB positivity rate was highest in lymph node sample (75% (6/8) followed by pus (57% (20/35), sputum (8.9% (149/1679) and gastric aspirate (7% (20/285). There was statistically significant association between Xpert MTB/RIF MTB positivity rate and sample types *(P*. *value* <0.001).

**Table 3:** Comparison of Xpert MTB/RIF assay results stratified by forms of TB (PTB and EPTB cases).

Sample type	Xpert MTB/RIF assay result						Total n (%)
	PTB-ND	EPTB-ND	PTB-D/RIF-S	EPTB-D/RIF-S	PTB-D/RIF-R	EPTB-D/RIF-R	
sputum	1530	0	146	0	3	0	1679(75.6)
Gastric aspirates	265	0	19	0	1	0	285(12.8)
pleural fluid	0	85	0	3	0	0	88(4)
cerebrospinal fluid	0	63	0	4	0	0	67(3)
Ascetic fluid	0	7	0	0	0	0	7(0.3)
Puss (abscess)	0	15	0	18	0	2	35(1.6)
lymph node	0	2	0	5	0	1	8(0.4)
Genitourinary	0	1	0	0	0	0	1(0.0001)
peritoneal	0	48	0	2	0	0	50(2.3)

Key; **PTB-ND-** PTB not detected; **EPTB-ND** EPTB not detected; **PTB-D/RIF-S** PTB detected, RIF sensitive **EPTB-D/RIF-S** EPTB detected, RIF sensitive **PTB-D/RIF-R** PTB detected, RIF resistance **EPTB-D/RIF-R** EPTB detected, RIF resistance.

### Detection of rifampicin resistant MTB

In total, rifampicin resistance was detected in seven (3.4%) patients by Xpert MTB/RIF assay. The frequency of rifampicin resistance was 9.5% and 6.7% among the new and previously treated cases, respectively. From a total of 171 pulmonary TB detected individuals, 2.3% (4/171) were resistant for rifampicin while 8.6% (3/35) were rifampicin resistance from extra-pulmonary TB group (Table 1). The prevalence of rifampicin resistance among the pediatric and adult TB patient’s was5.7% (2/35) and 2.9% (5/171) respectively. In HIV-positive patients, the prevalence of rifampicin resistant-MTB was 3% and in HIV-negative patients it was 4%.

## Discussion

In the current study, the overall prevalence of TB was found to be 9.3%. The result of this study was comparable to the previous study conducted in catchment area teaching health center over a five-year trend of 10.3% (showed a gradual decrease from 19.5% to 5.8% cases within five years) [15]. In contrast, the prevalence of TB in our finding was lower as compared to studies done in the other part of Ethiopia, including Gondar University Hospital, Northwest Ethiopia (24.6%) [16], Debre Markos Referral Hospital (23.2%) [17], Addis Ababa (15.11%) [18], Yirgalem hospital, Southern Ethiopia (16.5%) [19]. The possible explanations for the difference in the magnitude may be the difference in the study population, geographical variation, study setting, and TB control efforts such as the Directly Observed Treatment, Short Course program practices. In addition, sample heterogeneity could have a contribution to the Xpert MTB/RIF assay sensitivity.

In our finding, the prevalence of rifampicin resistance in EPTB patients was higher than in the PTB patients. A similar result was observed in a study conducted in Debre Markos Referral Hospital, Ethiopia [17]. However for detection of MTB, high yield of Xpert MTB/RIF assay was expected in pulmonary samples as compared to the extrapulmonary samples because of the paucibacillary nature of EPTB samples, which have a low bacterial load to be detected. This finding indicates that rifampicin-resistant extra-pulmonary *M*. *tuberculosis* infection becomes a major public health problem in high TB burden countries such as Ethiopia.

Another important finding was that the prevalence of childhood TB which is found to 6.3% in our study. In contrast to earlier findings from the same setting, surprisingly pediatric TB found to be about fivefold lower than the previous study (31.7%) [10] as well around two-fold lower than the study conducted in Addis Ababa (13.6%) [18]. The most important reason for the low prevalence of childhood TB in our study could be because of the sample types analyzed. It is well documented that the Xpert MTB/RIF assay detection rate is strongly affected by the types of the sample tested. In our study, a significant number of extrapulmonary samples and gastric lavages were tested by Xpert MTB/RIF assay. The other possible reason could be due to the fact that only Xpert MTB/RIF assay was used to diagnose childhood TB in in the current study whereas previous studies applied microscopy, culture, and clinical criteria to diagnose TB in children.

Furthermore, the current study found that the frequency of rifampicin resistance was 3.4%. This is similar with a study conducted in South Ethiopia (3.4%); however, prevalence PTB in their setting was two times higher [19]. In contrast, it was lower than a study conducted in other parts of the country; northwest Ethiopia 15.8% [16], Addis Ababa 9.9% [18], Bahir Dar and Debre Tabor 9.3% [20], and national survey 6.3% [21]. Our finding also accords with earlier observations in the same study setting, which showed a relatively low prevalence of first-line drug resistance in Southwest Ethiopia [22]. Many studies showed that rifampicin resistance is a surrogate marker for MDR-TB and WHO recommends that rifampicin-resistant-TB patients should be treated as patients with MDR-TB. The correspondence of sample type and Xpert^®^ MTB/RIF assay TB detection level is interesting because the yield of conventional TB diagnostic methods directly related to the types of the sample [3]. In the current study, the Xpert MTB/RIF assay detection rate of MTB was strongly associated to sample types; showing significantly high TB detection rate in puss (abscess) and lymph node samples supporting prior study [9] and in contrary poor yield in fluids (pleural, peritoneal, ascetic). These results match those observed in earlier studies [23–25]. The most likely causes of low yield in specified fluids related to the paucibacillary nature of the clinical samples and low bacterial load (less than 131CFU/ml sample, below the detection limit of Xpert MTB/RIF). However due attention needs to be given over the result due to unproportioned number of sample types used in the study.

Moreover, this study assessed different factors thought to be associated with TB. Of the factors assessed, age, previous TB treatment history, sample type, and peoples living with HIV were strongly associated with the detection level of MTB. These results match those observed in earlier studies [16–19]. In the present study, MTB yield was significantly higher among individuals living with HIV compared to naïve patients. This is in agreement with the recent WHO TB report; HIV as a major attributing risk factor of TB in sub-Saharan Africa countries besides most the gaps in regions was the detection and treatment of HIV associated TB [1]. The additional advantage of Xpert MTB/RIF assay over a conventional method is its improved yield in HIV infected individuals. Thus, patients living with HIV should undergo Xpert MTB/RIF assay testing for early diagnosis and treatment of TB.

The principal patient-related factor that is associated with the occurrence of drug resistant-TB is poor adherence to TB treatment. Previous studies reported that rifampicin-resistant MTB was significantly higher among previously treated patients compared to treatment naïve patients [18, 20]. In particular, those patients that have a previous TB treatment history such as treatment failures, defaulters, or relapse cases are at greater risk of developing drug resistant-TB. In the present study, we couldn’t see a statistically significant difference in rifampicin-resistant MTB among previously treated patients compared to treatment naïve patients. This could be because of the small number of rifampicin resistance cases reported in our study. Nevertheless, the presence of drug-resistant TB among new cases has significant public health importance. This indicates active transmission of drug-resistant MTB among the community and a new strategy should be in place to reduce the transmission of drug-resistant TB in the country.

The major strength of this study was the detection of *M*. *tuberculosis* and rifampicin resistance using Xpert MTB/RIF assay from a large sample size using respiratory and non-respiratory specimens in adults and children. However, our study was not without pitfalls. First, the study was retrospective and unproportioned sample types were included. Second; this study was conducted among patients seeking treatment at Jimma Medical Center, and the data may not represent the general population of Southwest Ethiopia. Thus, the finding of this study should be interpreted with these limitations.

## Conclusion

The overall frequency of MTB and rifampicin-resistance was 9.3% and 3.4%, respectively. Xpert MTB/RIF gave the highest yield in lymph node sample followed by pus and sputum. The study participants with the age group of 15-24-year, previous history of TB, specimen type (lymph node), and being HIV-positive were significantly associated with MTB positivity by Xpert MTB/RIF assay. These findings generally suggest that relatively lower burden of TB in study setting compared to previous studies conducted in Ethiopia. However, it is important to bear in mind that still huge gap presents with the proposed strategy by the government of Ethiopia to End TB by 2035, which targeted to reduce TB prevalence as low as 10 per 100,000 population. Although the frequency of TB disease is decreasing, more works should be done to further combat TB and drug resistant TB in the study area.

## Supporting information

S1 File The excel dataset used and analyzed in the current study. (XLSX)
